# Supplementary material for: A Novel Antimicrobial Peptide Sparanegtin Identified in Scylla paramamosain Showing Antimicrobial Activity and Immunoprotective Role In Vitro and Vivo
Source: Int J Mol Sci. 2021 Dec 21;23(1):15. doi: 10.3390/ijms23010015 (PMC8744658; doi:10.3390/ijms23010015)
Supplement: Supplementary file 1 [file ijms-23-00015-s001.zip › ijms-1487291-supplementary.pdf]

Supplementary materials

For

A Novel Antimicrobial Peptide Sparanegtin identified in *Scylla paramamosain* showing antimicrobial activity and immunoprotective role *in vitro* and *vivo*

Xuewu Zhu[1], Fangyi Chen[1,2,3], Shuang Li[1], Hui Peng[1,2,3], Ke-Jian Wang[1,2,3,*]

1State Key Laboratory of Marine Environmental Science, College of Ocean & Earth Sciences, Xiamen University, Xiamen, Fujian, China

2State-Province Joint Engineering Laboratory of Marine Bioproducts and Technology, College of Ocean & Earth Sciences, Xiamen University, Xiamen, Fujian, China

3Fujian Innovation Research Institute for Marine Biological Antimicrobial Peptide Industrial Technology, College of Ocean & Earth Sciences, Xiamen University, Xiamen, Fujian, China

* Correspondence: Ke-Jian Wang

College of Ocean & Earth Sciences, Xiamen University, Xiamen, Fujian 361102, PR China.

E-mail: wkjian@xmu.edu.cn (K.J. Wang)

Table and Figures

Table S1 Sequences of primers used in the study.

Primers	Sequence (5'-3')
*Sp*Toll2-qPCR-F	GTCCTCTCGTCCAGCTTCATTGAGA
*Sp*Toll2-qPCR-R	CAGGTGGGATCTTGCCATAGACAAT
*Sp*STAT-qPCR-F	TATATGGCTGAACCGGCACC
*Sp*STAT-qPCR-R	AGCAAGCGAGTGATGTGTGA
SpHyastatin-qPCR-F	TTACAACGCGAAGGTTCCGA
SpHyastatin-qPCR-R	GGCTTGGTGCCTACTCCAAT

*Sp*ALF2-qPCR-F	CGCGTGTCGATGCTTCTCGT
*Sp*ALF2-qPCR-R	ACCACACGTCTCCCCTGAAGT
*Sp*Myd88- qPCR-F	AGAATACGGGAACTGTCTGGGT
*Sp*Myd88-qPCR-R	TGGTGACTGTGATGGAGATGGT
*Sp*CAT-qPCR-F	CAACACTCCCATCTTCTTCATCAGG
*Sp*CAT-qPCR-R	TGTTGTTTCTGGACGCAGGGTGAT
*Sp*SOD-qPCR-F	GGGGATGGGAAACAACTCTGGAT
*Sp*SOD-qPCR-R	GGTGCCTTGGTTAAATACACGGTGC
*Sp*GPx-qPCR-F	GCAACCCAGTCAAGAGATTCAGC
*Sp*GPx-qPCR-R	AGGATGCAAGGAGGTGCTTCTG

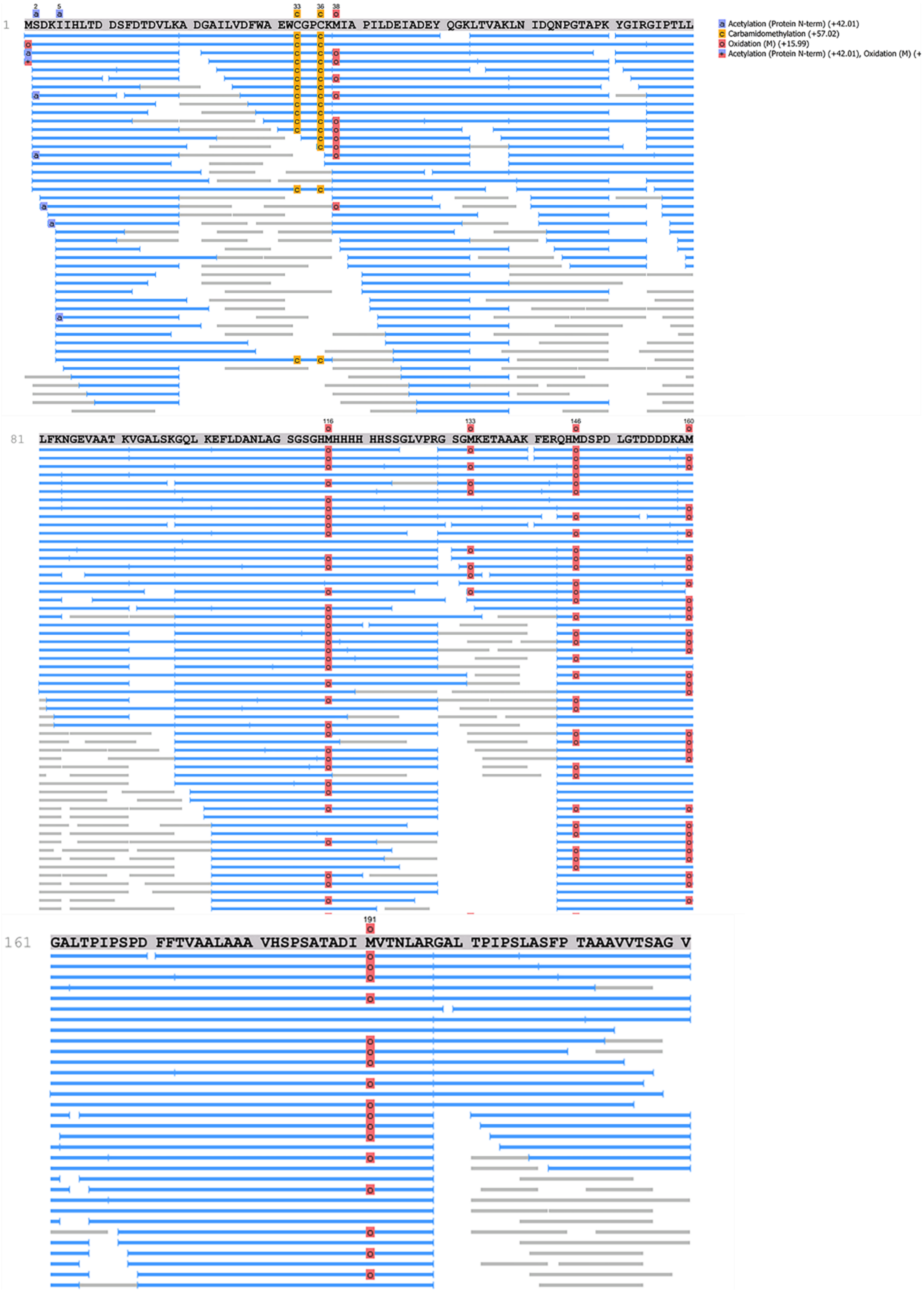

Figure S1 Mass spectrometry analysis of rSparanegtin.
